# Using ^18^F-FDG PET/CT to rule out Richter transformation as cause of deterioration in a patient with chronic lymphatic leukemia and severe COVID-19: A case report

**DOI:** 10.1097/MD.0000000000027545

**Published:** 2021-11-05

**Authors:** Lasse Fjordside, Helene Mens, Ali Asmar

**Affiliations:** a Department of Infectious Diseases, Rigshospitalet, Copenhagen University Hospital, Copenhagen, Denmark; b Department of Clinical Physiology, Nuclear Medicine & PET, Rigshospitalet, Copenhagen University Hospital, Copenhagen, Denmark.

**Keywords:** flourine-18 fluorodeoxyglucose positron emission tomography/computed tomography, chronic lymphatic leukaemia, COVID-19, Richter syndrome, Richter transformation, severe acute respiratory syndrome coronavirus 2 infection

## Abstract

**Rationale:**

This case report demonstrates the use of flourine-18 fluorodeoxyglucose (^18^F-FDG) positron emission tomography (PET)/computed tomography (CT) to rule out Richter transformation (RT) as the cause of clinical deterioration in a patient with chronic lymphatic leukemia (CLL) and severe COVID-19. ^18^F-FDG PET/CT can be used to establish the diagnosis of RT in patients with CLL, but the use of ^18^F-FDG PET/CT to exclude RT as the cause of clinical deterioration in patients with CLL and severe COVID-19 has not previously been described.

**Patient concerns:**

A 61-year-old male with CLL and COVID-19 developed increased dyspnea, malaise and fever during hospitalization for treatment of severe and prolonged COVID-19.

**Diagnoses:**

^18^F-FDG PET/CT ruled out RT and revealed progression of opacities in both lungs consistent with exacerbation of severe acute respiratory syndrome coronavirus 2 pneumonia.

**Interventions:**

^18^F-FDG PET/CT imaging.

**Outcomes:**

The patient was discharged at day 52 without the need of supplemental oxygen, with normalized infection marks and continued care for CLL with venetoclax.

**Lessons:**

^18^F-FDG PET/CT ruled out RT as the cause of deteriorations in a patient with CLL and severe COVID-19, enabling directed care of exacerbation of severe acute respiratory syndrome coronavirus 2 pneumonia.

## 1 Introduction

Patients with chronic lymphatic leukemia (CLL) are at increased risk of severe COVID-19, likely due to dysfunctional lymphocytes.^[1]^ During the course of severe COVID-19, some patients experience relapses and lack of treatment response. In these situations, distinguishing between hospitals acquired super-infections, relapse of viral replication or immunological hyperactivity as underlying causes is essential when determining the treatment strategy. While this may often proof to be a challenging task in itself, treating patients with CLL and severe COVID-19 is further complicated by the risk of Richter transformation (RT) that also has to be considered as the potential cause of sudden deteriorations.

RT refers to the change of indolent CLL to aggressive non-Hodgkin lymphoma.^[2]^ Patients typically present with fever (in the absence of infection), progressive lymph node enlargement, and elevated lactate dehydrogenase. The risk of transformation is independent of disease stage, duration or response to prior treatment, and may be triggered by viral infections (eg, Epstein–Barr virus), which are common in immunocompromised patients.^[3]^ Transformation occurs in about 5% to 16% of patients with CLL and has a poor prognosis; median survival with conventional chemotherapy is less than 1 year.^[4]^ Flourine-18 fluorodeoxyglucose (^18^F-FDG) positron emission tomography (PET)/computed tomography (CT) imaging has been used to identify such transformation; the absence of foci with significant ^18^F-FDG uptake can exclude the diagnosis of RT with a high degree of confidence,^[5]^ thus ^18^F-FDG PET/CT could be a useful tool in ruling out RT in CLL patients with severe COVID-19.

## 2 Case

**Figure 1:**
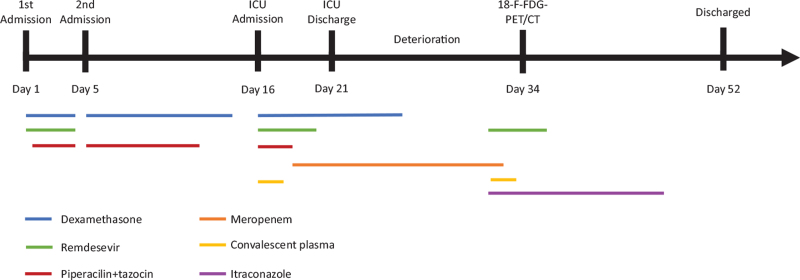
The course of time during hospitalization.

A 61-year-old Caucasian male known with a difficult to treat variant of CLL (TP53pos 13q mutated) and accompanying B-cell paresis presented to the ED with fever and general malaise was admitted with a SARS-CoV2 positive pneumonia with the need of 4 L/min nasal oxygen supply. The course of time during hospitalization is shown in Figure 1.

The patient had received treatment with ibrutinib for 6 months before admission, but this had been stopped due to hematuria and subcutaneous bleeding. In addition, the patient had received 2 courses of rituximab, fludarabine, and cyclophosphamide before admission together with intravenous gamma globulin.

**Figure 2:**
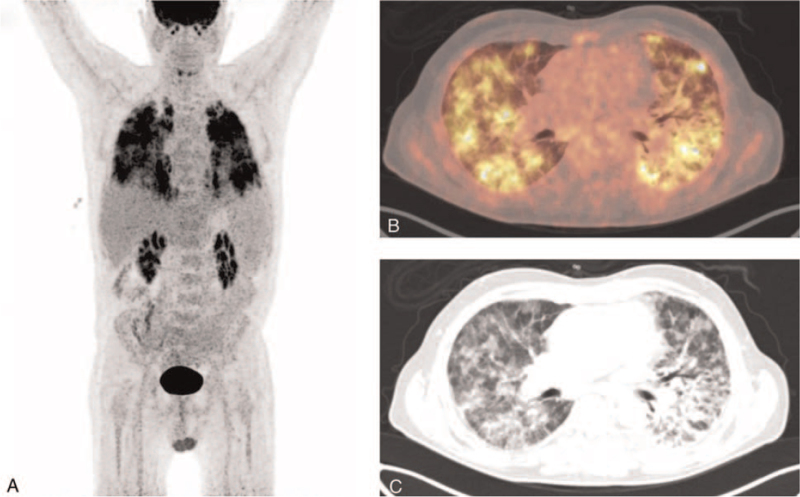
PET MIP (A), axial fused PET and contrast-enhanced CT images (B), and contrast-enhanced CT images. ^18^F-FDG = flourine-18 fluorodeoxyglucose, CT = computed tomography, PET = positron emission tomography.

The patient received treatment with remdesivir (200 mg on day 1 and 100 mg the following days i.v.)^[6]^ and oral dexamethasone (6 mg daily)^[7]^ for 4 days and was discharged due to bettering of respiratory symptoms. However, the patient was readmitted within 24 hours with fever and respiratory deterioration and oxygen need of 10 L/min. He was re-treated with dexamethasone and broad-spectrum antibiotics. Due to respiratory worsening, treatment with dexamethasone was prolonged to a total of 14 days. Convalescence plasma was added on admission day 16 along with a second course of remdesivir of 4 days. On admission day 16, the patient was transferred to the intensive care unit because of further respiratory worsening. Dexamethasone was re-initiated, the patient stabilized respiratory on high-flow oxygen without the need for intubation and was back in the COVID ward admission day 21. Due to continuous fever and increasing inflammatory parameters, itraconazole (200 mg twice daily), convalescence plasma and remdesivir was initiated on day 30. At this point of the admission, transformation of CLL and opportunistic disease were considered as co-factors for not getting better. The patient refused bronchoalveolar lavage and bronchoscopy, however ^18^F-FDG PET/CT imaging was carried out (Fig. 2).

PET MIP (Fig. 2A) and axial fused PET and contrast-enhanced CT images (Fig. 2B) demonstrated inhomogeneous high ^18^F-FDG uptake corresponding to diffuse ground glass and consolidative opacities in both lungs consistent with SARS-CoV2 infection (Fig. 2C).^[8,9]^ Nonenlarged mediastinal and iliac lymph nodes with slightly increased ^18^F-FDG uptake were also demonstrated and likely related to the patient's CLL. PET/CT images did not reveal any signs of transformation.

The ^18^F-FDG PET/CT scan allowed clinicians to continue care directed toward progressive pulmonary involvement of SARS-CoV2 infection according to guidelines. The patient adhered to and tolerated the treatments well. The patient was discharged to his own home at day 52 without the need for supplemental oxygen and with normalized blood-works. He continued care for CLL with venetoclax^[10]^ and was enrolled in an outpatient post-COVID follow-up program.

## 3 Discussion

Patients with CLL are at increased risk of severe COVID-19^[1]^ and during the course of disease, patients may experience deteriorations and lack of treatment response. RT can potentially be the cause of such sudden set-backs or lack of treatment response in patients with CLL and severe COVID-19, but distinguishing RT from progression of the severe acute respiratory syndrome coronavirus 2 infection or other opportunistic infections is challenging. The use of ^18^F-FDG PET/CT to detect RT in patients with CLL is well established,^[5]^ but the usefulness in excluding RT during severe COVID-19, has not, to our knowledge, been proposed before.

Although PET/CT offers no clinically significant additional value in the standard diagnostic management of COVID-19,^[11]^ it should be considered in patients with CLL and deteriorations during standard treatment regimens for severe COVID-19 in order to rule out RT as the cause.

## 4 Limitations

As previously described the patient refused bronchoscopy and bronchoalveolar lavage which made ruling out opportunistic infections as cause of the clinical and para-clinical deterioration impossible.

Using the cycle threshold value from daily severe acute respiratory syndrome coronavirus 2 PCRs as an invert proxy for viral load was not enrolled in clinical practice in our hospital at the time the case took place, but could potentially have aided the differentiation between COVID-19 progression and other causes for the clinical worsening. This resulted in a more broad and aggressive treatment strategy and thus an increased risk of significant side effects and the obvious loss of a clear therapeutic target making withdrawal of individual treatments more difficult.

## Author contributions

**Writing – original draft:** Lasse Fjordside, Helene Mens, Ali Asmar.

**Writing – review & editing:** Lasse Fjordside, Helene Mens, Ali Asmar.

## References

[1] Mato AR Roeker LE Lamanna N. Outcomes of COVID-19 in patients with CLL: a multicenter international experience. Blood 2020;136:1134–43. 32688395 10.1182/blood.2020006965 PMC7472711

[2] Omoti CE Omoti AE. Richter syndrome: a review of clinical, ocular, neurological and other manifestations. Br J Haematol 2008;142:709–16. 18492119 10.1111/j.1365-2141.2008.07248.x

[3] Rossi D Spina V Gaidano G. Biology and treatment of Richter syndrome. Blood 2018;131:2761–72. 29692342 10.1182/blood-2018-01-791376

[4] Allan JN Furman RR. Current trends in the management of Richter's syndrome. Int J Hematol Oncol 2018;7:IJH09. 30651968 10.2217/ijh-2018-0010 PMC6331753

[5] Bruzzi JF Macapinlac H Tsimberidou AM. Detection of Richter's transformation of chronic lymphocytic leukemia by PET/CT. J Nucl Med 2006;47:1267–73. 16883004

[6] Beigel JH Tomashek KM Dodd LE. Remdesivir for the treatment of Covid-19 – final report. N Engl J Med 2020;383:1813–26. 32445440 10.1056/NEJMoa2007764 PMC7262788

[7] Group RC Horby P Lim WS. Dexamethasone in hospitalized patients with Covid-19. N Engl J Med 2021;384:693–704. 32678530 10.1056/NEJMoa2021436 PMC7383595

[8] Ai T Yang Z Hou H. Correlation of Chest CT and RT-PCR testing for coronavirus disease 2019 (COVID-19) in China: a report of 1014 cases. Radiology 2020;296:E32–40. 32101510 10.1148/radiol.2020200642 PMC7233399

[9] Qin C Liu F Yen TC. (18)F-FDG PET/CT findings of COVID-19: a series of four highly suspected cases. Eur J Nucl Med Mol Imaging 2020;47:1281–6. 32088847 10.1007/s00259-020-04734-w PMC7080035

[10] Hallek M. Chronic lymphocytic leukemia: 2020 update on diagnosis, risk stratification and treatment. Am J Hematol 2019;94:1266–87. 31364186 10.1002/ajh.25595

[11] Fields BKK Demirjian NL Dadgar H. Imaging of COVID-19: CT, MRI, and PET. Semin Nucl Med 2021;51:312–20. 33288215 10.1053/j.semnuclmed.2020.11.003 PMC7703471

